# Financialising acute kidney injury: from the practices of care to the numbers of improvement

**DOI:** 10.1111/1467-9566.12868

**Published:** 2019-02-12

**Authors:** Simon Bailey, Dean Pierides, Adam Brisley, Clara Weisshaar, Thomas Blakeman

**Affiliations:** ^1^ Centre for Health Services Studies University of Kent Kent UK; ^2^ University of Stirling Stirling UK; ^3^ University of Bristol Bristol UK; ^4^ Manchester Business School, University of Manchester Manchester UK; ^5^ Centre for Primary Care Institute of Population Health Manchester UK

**Keywords:** Finance, funding, budgeting, Health service organisations, political, quality of care, safety, ethnography

## Abstract

Although sociological studies of quality and safety have identified competing epistemologies in the attempt to measure and improve care, there are gaps in our understanding of how finance and accounting practices are being used to organise this field. This analysis draws on what others have elsewhere called ‘financialisation’ in order to explore the quantification of qualitatively complex care practices. We make our argument using ethnographic data of a quality improvement programme for acute kidney injury (AKI) in a publicly funded hospital in England. Our study is thus concerned with tracing the effects of financialisation in the emergence and assembly of AKI as an object of concern within the hospital. We describe three linked mechanisms through which this occurs: (1) representing and intervening in kidney care; (2) making caring practices count and (3) decision‐making using kidney numbers. Together these stages transform care practices first into risks and then from risks into costs. We argue that this calculative process reinforces a separation between practice and organisational decision‐making made on the basis of numbers. This elevates the status of numbers while diminishing the work of practitioners and managers. We conclude by signalling possible future avenues of research that can take up these processes.

## Introduction

In the wake of the 2008 credit and sovereign debt crises, scholars have sought to understand how financial concerns increasingly shape economies and states (Guillén and Suárez 2010, Perrow 2010, Stiglitz 2010). It is retrospectively argued that the process now known as ‘financialisation’ can be traced back to the 1960s crises of state, experienced first in the US, then spreading to the UK, parts of Europe and Australia over the next decade. At this time, excessive government debt, balance of payment problems and incompatible social demands led to a loss of public confidence in the state's ability to manage these problems (Krippner 2012). In response, conventional paradigms of public administration gave way to ‘new’ managerial discourses (Hoggett 1996, Hood 1991, Pollitt *et al*. 1991). Various forms of marketisation were also promulgated to purportedly increase efficiency, flexibility and liberation from bureaucratic ties (e.g. Clegg *et al*. 2011). Sociological literature that focuses on health care provides rich descriptions of these processes and draws our attention to the various effects of management and markets upon practices and processes of care (e.g. Mol 2008, Moreira 2013), as well as upon the work, identities and ethics of health practitioners and managers (e.g. Noordegraaf 2011, Waring and Currie 2009). Financialisation finds its roots in these same processes but it disturbs the conventional coupling of management and markets, resulting in organisational practices that exceed our extant understandings of managerialism in healthcare and other public sectors. Similarly, while the production and circulation of numbers has been shown to be an important part of a neoliberal apparatus of government (Hacking 1990, Rose 1999), financialisation encourages us to look at the specifically financial character of politicisation that we find in contemporary modes of government, as this shapes decisions about the kind of numbers to produce and the purposes to which they are put.

In this study, we build on the public sector financialisation literature (Froud *et al*. 2009, du Gay *et al*. 2012) to draw attention to the processes of abstraction, measurement and tabulation which are generated in qualitative knowledge practices through the deployment of technologies and programmes ostensibly concerned with patient safety and quality improvement (QI). Our argument seeks to extend existing sociological enquiry into one of the *dilemmas* of quality and safety practice: epistemologies which abstract and concretise in an attempt to reduce everyday complexity and make it measurable (Waring 2013). Following Waring (2009), we are interested in how this process results in the generation of ‘sociocultural and political dynamics of organisational life’ that can be analysed in order to better understand the practices they shape. Financialisation provides an underpinning logic to this process, but it also alerts us to a set of problems to do with the detachment of quantitative measures from the qualitative practices out of which they are generated, and the effect this has in situating an alternative way of thinking and doing within practice. In our argument, financialisation – through new organisational technologies which map, quantify and automate – brings into effect a set of organisational arrangements for establishing debt and investment. Numbers can then displace, or hijack, other kinds of values such as quality or safety. Our focus on the relationship between what we refer to as ‘qualitative’ knowledge and practice, which we contrast with the ‘quantitative’ bundling of these practices, is part of an attempt to demonstrate how numbers are created and become authoritative with regards to the practices out of which they are produced. This effects what we refer to as an organised separation between the domain of healthcare practices and the numbers assembled in those practices, with the qualitative domain, not only reduced via its abstraction to the quantitative but also diminished within that domain due to the status of numbers as ‘undisputed future projections’ (Mazmanian and Beckman 2018). Financialisation is becoming a more mainstream term within studies of political economy, however, it is still on the margins of sociological discourse and to date there is only a single sociological study of financialisation in health care, which examines the effects of private equity funds in the Turkish health system (Vural 2017). The organisation of these processes within publicly funded health systems has not yet been subjected to scrutiny within the medical sociological research literature.

We draw on ethnographic data from a study of a QI programme in an acute hospital trust providing specialist renal services in the National Health Service (NHS) in England, in order to set up a broader discussion of financialisation in health care. The data set reported here comprises observational, interview and documentary data collected over a period of approximately 2 years. The data were generated during the set up and implementation of a QI programme for acute kidney injury (AKI), a syndrome characterised by a sudden reduction in kidney function.

In 2014–15, the national commissioning body for the NHS in England promoted the organisational attention given to AKI through two programmes: first, they issued a patient safety alert which mandated the introduction of an algorithm into all NHS laboratories to standardise the identification of AKI (NHS England, 2014); second, they launched a 12th month incentive programme, which was aimed at improving the information recorded and documented about AKI on patient discharge summaries (NHS England, 2015). These two programmes shaped the core focus of the QI work undertaken in our empirical site. They also signalled to us two different ways in which qualitative practices could be rendered smooth and calculable. Our task then became one of seeing, firstly, how this *calculus‐like logic* could take hold within a domain governed by the knowledge practices of medical experts (Aveling *et al*. 2016); secondly, through what ‘frictions and flows’ the translation of qualitative into quantitative could take place (Fiore‐Silfvast 2014) and lastly, the effects of this transformation upon the decisions and actions of the managers and professionals responsible for its implementation, and whose work would is consequently be ‘taken into account’ (Allen 2016).

We argue that once these translations have occurred, the generated numbers become organisationally detached from the ethical, social and political aspects of experience out of which they were assembled, yet they continue to participate in that experience, but on new terms. By subsequently forming the basis of financial decision‐making within and across organisations, these numbers end up bearing down on the very practices out of which they were produced. To build this argument we describe three processes from our empirical research through which this occurs: (1) representing and intervening in kidney care; (2) making caring practices count and (3) decision‐making using kidney numbers. This three‐part mechanism is one in which care practices are rendered problematic and worked upon through organisational technologies which map, quantify and automate. The assumptions embedded in the attempt to make practice calculable is that it can be made predictable, and in a sense *perfectable*. Working with these assumptions, it becomes possible to rate care practices according to their states of non‐compliance and to control them on new terms.

Financialisation represents a new frontier for exercising organisational control in public organisations, created by a shift in the activities of frontline practitioners and managers towards the assembly and maintenance of financial numbers. Through ethnographic data we attend to the means by which ‘quality’, ‘safety’ and ‘improvement’ find expression within daily care practices through the mobilisation of specific mechanisms for each process we describe. In our study of AKI these are operationally achieved through the introduction of methods and materials of improvement such as process mapping, algorithms, electronic patient records (EPR) and incentive payments. These methods and materials are in common use in improvement programmes in health services internationally, underscored by strong institutional support (e.g. Institute of Healthcare Improvement, 2003). Our interest in these organisational technologies is the work they do in bringing into effect a new decision‐making repertoire for interrogating practice using numbers that participate in debt and investment. This extends research on commodification in health care, for example, through pay for performance frameworks (Checkland *et al*. 2007, Norman *et al*. 2016) because it sets up a future *financial* state of affairs and establishes debt relations between this anticipatory future and everyday practice, alongside an apparatus for measuring and recording practice ‘improvement’. Together these mechanisms enact a series of translations, from care to risk and from risk to cost. The process involves the separation of numbers from the knowledge practices that have produced them and a subsequent relocation of these numbers to a different domain. In this new location, these numbers can then accomplish two kinds of work: intervening ‘back’ upon and shaping care practices, and presenting public accounts of the organisation, by being mobilised ‘out’ in order to shape future decisions made by and on behalf of the organisation. In a context of austerity, such decisions can threaten the organisation's very existence. In this same vein, financialisation effects a shift in extant understandings of managerialism in public organisation (e.g. Ferlie *et al*. 1996). As numbers are separated from practices, the activities of frontline practitioners, managers and other skilled knowledge workers increasingly focus on the maintenance of those numbers which uphold organisational narratives (Cushen 2013), simultaneously eclipsing everyday struggles to create and maintain improvement.

To introduce financialisation to medical sociology we begin with a detailed description of it as a concept. We first situate it in the macroeconomic conditions of the 1970s. Then we outline its relationship to neoliberalism and public policy, before moving towards meso‐ and microlevel financialisation and the production of numbers through the work of frontline practitioners and managers, where our own analysis begins.

## Financialisation in economy, society and (public) organisation

Financialisation is a term used to describe the ‘increasing role of financial motives, financial markets, financial actors and financial institutions in the operation of the domestic and international economies’ (Epstein 2005). This increasing role signals a shift in the meaning and consequences of accumulation and value in capitalist systems. Finance has always played an important part in capitalist systems of accumulation, as the source of capital through which productive capacity could be increased and from which greater profit could be derived. This role is embedded in capitalism through the circulation of commodities, money and credit. The vastly expanded role of finance observed in Anglo‐American and latterly global capitalism since the 1970s marks a deepening and broadening of this embedding (Fine 2010). This has led to the proliferation of increasingly complex and future‐oriented derivative forms of finance, described collectively as ‘fictitious capital’ (Haiven 2014). The result is a shift away from production as the heart of capitalist accumulation through the ‘elimination of productive capacity and employment’ (Rossman and Greenfield 2006), with profit no longer derived from growth in the fixed capital means of production, but from the extraction of value, specifically shareholder value. Ownership then no longer means controlling the means of production, as a Marxist analysis would have it, but instead control of technology and information which guides short‐term financial restructuring and outsourcing to maximise returns, hence Lazonick and O'Sullivan's (2000) claim that the modus operandi of capitalism has shifted under financialisation from ‘retain and reinvest’ to ‘downsize and distribute’. This signals a shift from value creation to ‘value capture’ (Krippner 2005), with companies reduced to a ‘bundle of assets’ to be traded (Froud and Williams 2007).

Although it is recognised that financialisation does not represent a single or unitary ‘logic’, nevertheless there is a broad consensus over both the conditions that have led to, and the consequences of, the contemporary experience of financialisation (Froud *et al*. 2007). Initiated by the policy response to the US dollar crisis in the 1970s, financialisation took root in the new possibilities created by international currency trading and the ‘floating’ exchange rate (D'Arista 2005).

In the UK, the experience of financialisation is intimately tied to neoliberalism, as the political polarisation that was caused by the economic decline in the 1970s eventually resulted in the election of the Conservative Government in 1979, which led to the implementation of a radical agenda, beginning with ‘experiments with privatisation and deregulation, supply side policies and aspirations for a smaller state’ (Gamble 2009). Deregulation had the effect of expanding the scale and scope of markets for currency futures, which then moved into derivatives, leading in turn to the rise of ‘pension fund capitalism’ and a ‘revolution in retail banking’, which turned citizens into (somewhat unwitting) consumers of financial products (Froud *et al*. 2007). The neoliberal values of privatisation and self‐responsibility therefore find their monetary expression in financialisation; thus, finanicalisation becomes the individual pursuit of self‐interest as the means to (financial) freedom (Langley 2004, c.f. Rose 1999). This demands ‘new identities and forms of calculation’ from citizens, without the knowledge and technology with which to make ‘sensible choices’ (Froud *et al*. 2007).

Nevertheless, the relationship between financialisation and neoliberalism is a complex one, and this can be examined through the changing role of the state vis‐à‐vis the market. As the financial sector comes to dominate the productive sector, national economic sovereignty is undermined and a marketisation of financial relations leads to the ‘disembedding of global finance from national programmes of governance’ (Froud *et al*. 2007). This undermines the role of the state as an active agent of economic restructuring; hence, the neoliberal relationship between financial deregulation and the shrinking role of the state. In turn, the role of the state in social policy and welfare is eroded. At the same time the state becomes an active agent of finance capitalism; beginning in the 1970s, as noted above, there is state intervention through economic policy that promotes the interests of private capital and financial markets. This is what Fine (2010) refers to as the ‘first phase’ of financialised neoliberalism. The second phase is marked by state intervention to ‘moderate the negative impact of finanicalisation’ (Fine 2010). This began with the credit crisis of 2007 which precipitated the sovereign debt crisis caused by the public bail out, first of Northern Rock and subsequently of RBS, HBOS and Lloyds. For some, this represents a shift away from one strand of neoliberalism in the retention of a strong (albeit altered) state role in economy and society, for others it represents the enrolment of the state as a mediator of global financial interests through domestic economic and social policy (Fine 2010, Fine 2012).

The cross‐party neoliberal consensus that can be observed through the trajectory of public policy in the UK over the past three to four decades has become fused in the contemporary experience with the sovereign debt crisis. This has diverse consequences; most obviously austerity spending, privatisation and outsourcing, but also de‐politicisation through the creation of semi‐autonomous and ‘arms‐length’ regulatory bodies and quasi‐corporate structures, and the dissemination of investment and incentive schemes which act as ‘hinges’ through which the interests of private capital can access the balance sheets of public organisations directly (du Gay *et al*. 2012).

Studies of financialisation at the organisational level have emphasised the role of strategic narratives produced between top management and external financial actors in order to construct future‐oriented stories of value creation (Erturk *et al*. 2008, Froud *et al*. 2006). Management narratives are optimistic and characterise the organisation as pursuing strategies through which to achieve future returns. These narratives shape and are shaped by the valuation and investment decisions of other actors, creating a sense of order and stability in the inherently uncertain and unstable financial market. The task of senior management is then to ensure that there are numbers with which to corroborate narratives (Hendry *et al*. 2006).

Extending this position, Cushen (2013) argues that financialisation at the microlevel is ‘defined precisely by the stream of performative interventions organisations take to live the narrative’. Narrative construction empowers senior management to set their own performance targets, which are then ‘sold’ to ‘the market’ (Hendry *et al*. 2006). Further down the hierarchy the task of managers and other knowledge workers becomes a practice of assembling the numbers in order to maintain the narrative (Cushen 2013). This elevates the role of accounting in organisations and at the microlevel brings into view techniques and practices of accounting (Bresnen *et al*. 2017). These techniques and practices are not neutral or value free but rather represent the attempt to ‘quantify and compare things which, by their very nature, are neither quantifiable nor directly comparable’ (Perry and Nölke 2006). This is the core concern we explore through our ethnographic data. We examine how financial projections and narratives play a key role in the representation and problematisation of practices, which are transformed first into risks, and then into costs.

## Acute kidney injury

Our study draws on ethnographic data from a study of a QI programme for AKI in an acute hospital providing specialist renal services for an urban population of around 1.5 million people. AKI is a syndrome characterised by sudden and potentially lethal damage to the kidneys, often as a secondary product of infection, dehydration or medication, which affects between 5–15% of all hospital admissions in England (Kerr *et al*. 2014). The problems that are now associated with the term ‘AKI’ were first identified from autopsies of wounded soldiers in World War II and furthered through observations made during the Korean war, which concluded that post‐traumatic renal inefficiency was a major cause of death among the severely wounded (Bywaters and Beall 1941, Teschan *et al*. 1955). The term AKI is more recent, having first been described in 2004 as part of an attempt to generate consensus over the clinical definition and diagnostic criteria for acute renal failure (Bellomo *et al*. 2004). It is only in the wake of a highly critical report by the National Confidential Enquiry into Patient Outcome and Death (NCEPOD, 2009), subsequent national and international guidelines (KDIGO, 2012, NICE, 2013), and an economic analysis suggesting substantial underestimation of the prevalence and cost of AKI (Kerr *et al*. 2014) that AKI has emerged as an improvement priority for English healthcare organisations.

In 2014, the national NHS commissioning body issued a patient safety alert aimed at standardising the early identification of AKI. It mandated, within all NHS laboratories, the introduction of an algorithm which would identify potential cases of AKI from laboratory data, and produce a result which could then be communicated through IT patient management systems. All laboratory systems were expected to ‘go live’ by March 2015. At the same time, NHS England put out a commissioning directive for AKI known as a CQUIN (Commissioning for Quality Improvement; hereafter, incentive scheme), which sets out particular conditions that if satisfied by the organisation adopting them results in a bonus payment. The AKI incentive scheme (NHS England, 2015) required that patient discharge summaries include four items:

1. Stage of AKI (a key aspect of AKI diagnosis);
2. Evidence of medicines review having been undertaken (a key aspect of AKI treatment);
3. Type of blood tests required on discharge for monitoring (a key aspect of post discharge care);
4. Frequency of blood tests required on discharge for monitoring (a key aspect of post discharge care)

These incentive schemes are not mandatory, and the organisations adopting them can fix their own targets and compliance levels within given parameters.

## Research methods

Our study was part of a larger project which sought to use ethnographic methods to explore in‐depth the implementation and spread of QI programmes related to AKI in secondary care. The broader aim was to develop a nuanced organisational account of the nature and role of context in QI programmes, by examining the material accomplishment of key values such as ‘quality’, ‘improvement’ and ‘safety’, also attending to ways in which the often informal work of improvement could be made more visible.

The study was set up and funded as part of the National Institute of Health Research (NIHR) Collaboration for Leadership in Health Research and Care (CLAHRC) programme. CLAHRCs are research networks involving close partnerships between research and practice in the NHS. The trust in which our study took place was an existing partner of the network, and the study scope emerged from a series of conversations between the researchers and the QI team in the trust initiated in March 2015. The protocol was agreed, and internal ethical review was completed in July 2015, at which point the research team started observing programme meetings. A full ethics application was also initiated at this time and received a favourable opinion from the Wales 7 REC committee (15/WA/0400, 16th November 2015).

The data set comprises observational, interview and documentary data collected over a period of 24 months. The data were generated during the planning, set up and implementation of the first phase of the QI programme and the incentive scheme implementation period. At the conclusion of data collection the programme had completed the first ‘initiation’ phase and had started the second ‘spreading’ phase.

Observations were made of meetings and other events associated with the QI programme itself as well as time spent observing routine activities on wards. The programme itself was made up of a series of learning events (one whole day and four half day events over a 12‐month period), attended by representatives of each of the 11 wards involved in the programme. These learning sessions were comprised of three main elements: (1) learning about improvement methodology; (2) learning about AKI and (3) setting and monitoring targets for improvement. Over time, the emphasis shifted towards the ongoing monitoring of targets, which occurred partly through the statistical run charts displayed presentation style by one of the QI team, and partly through the more interactive development of ‘tests of change’ (see ‘Quality Improvement Methodology’, below). These tests were then tried out through routine activities on the wards, fed back to subsequent learning sessions and refined over time.

In addition to the learning sessions for the QI programme, regular meetings were observed involving members of the QI team along with a wider steering group made up of key clinical and managerial staff from across the hospital, including divisional management, IT specialists, acute, surgical and renal consultants, and learning and development coordinators. This group met on average approximately once per month throughout our study.

Interviews were conducted with members of the three main groups that comprised the improvement work in the hospital: the QI team, the steering group and the clinical teams participating in the QI programme. Interview schedules were unstructured and were intended to elaborate upon themes identified through the ongoing observational work.

## Quality improvement methodology

The trust we describe here had a specific directorate responsible for QI, which had adopted the Institute of Health Improvement's ‘break through series’ approach to QI. This is a collaborative approach which aims to achieve sustainable improvement through ‘culture change’ (Institute of Healthcare Improvement, 2003). Programmes are made up of a series of learning sets which involve frontline workers from across relevant wards and departments, usually around four sessions over a 1‐year period. During these sessions staff are instructed in improvement methodology, which draws on a systems approach to organisation and management, informed particularly by safety science. Staff are then asked to design ‘tests of change’ through which they aim to make improvements in their respective wards and departments. In between each learning session these ‘tests’ are carried out and the results and reflections brought to the next learning session. The overall objective of the learning sessions and tests of change is to generate a ‘change package’, with concrete steps for the achievement of the desired change, which can then be spread within and across the relevant wards and departments. The hospital had also adopted the incentive scheme for AKI, and from March 2015 onwards had to submit quarterly data excerpts to NHS England. In contrast to the collaborative and developmental approach of the QI programme, the incentive scheme was seen by the programme managers as a ‘top down’ and ‘tick box’ exercise.

Acute kidney injury is understood in national and international policy and guidance as a safety problem, with improvement aimed at reduction in preventable harm. Although safety has been a core concern of organised medicine for many years, it has risen to prominence in the past two decades, and has become almost synonymous with the idea of quality – such that quality in health care can be measured as compliance with standardised tools and methods such as checklists and audits (Waring *et al*. 2016). This doctrine reached a kind of zenith in the dissemination by the leading US Institute of Health Improvement of what it called the ‘triple aims’, according to which all QI programmes should simultaneously strive to achieve improvements in ‘care, health and cost’ (Berwick *et al*. 2008). Via the doctrine of safety, quality in health care therefore becomes intimately tied with waste and efficiency. Under austerity this increasingly becomes pecuniary efficiency.

The QI programme we observed took place in a context that faced extreme pressures due to the austerity spending programme first implemented in 2010, which continues to the present. Over the period of our observations within the hospital, the QI team faced a constant battle attempting to engage staff in the programme, when this meant leaving shortages on wards which had experienced severe reductions in staffing numbers amid rising levels of demand. It is within this context that the promise of a syndrome like AKI, with its preventable harms, cost savings and incentive schemes, is made an attractive object of intervention. However, intervention on these terms resulted in a close and problematic coupling of QI with cost saving, which created ongoing management challenges throughout the duration of the programme.

## Findings

### Representing and intervening in kidney care

The formation of the object AKI requires the context in which AKI is experienced to first be made visible in a way which renders the everyday, tacit experience of the organisation problematic. We draw on the empirical example of process mapping to illustrate this. Process mapping was part of the initial setup of the quality and safety programme. It was initiated by the QI team. The objective of the process map was to develop a visual representation of what happens to a patient upon entering the clinical area or ward being mapped. On this occasion, the area being mapped was the Emergency Assessment Unit (EAU). In the following excerpt, the EAU manager began facilitating the process mapping in the absence of the QI manager who had not yet arrived. An EAU consultant was present, as were three or four ward staff – nurses and health care assistants.

The mapping proceeds with the manager asking prompting questions and then writing down the responses of the clinical staff on coloured notes and sticking them on a white board to make the map. The manager opens with the question and then follows up responses with further prompts, in the following manner (Note; not verbatim transcript):

Q: How do AKI patients get onto the system
A: Elective or emergency admissions.
Q: Where do emergency admissions come from
A: A&E, primary care, EAU, specialist referral
Q: Tell me about EAU
A: All patients are seen and assessed, and a risk assessment is carried out
Q: What factors are considered?
A: Depends on the patient, and the kind of admission, there are many different possible risk factors you might look at.
Q: What happens next?
A: Bloods are taken
Q: And sent to biochemistry?
A: Not always, depends on severity

At this point the exercise was <5 min old, and there was a lot of discussion, and some debate and confusion about how the sticky notes should be placed in relation to one another. Some problems challenged the format of the sticky notes, some elements of the care process, such as the risk assessment that took place when patients first entered EAU, were too multifactorial and context dependent to be represented in this way. It was not practical to have a different note for each different possible risk factor, and yet, different risks prompted different possible actions. The ideal of the linear pathway that can be mapped was being challenged. The exercise continued:

Around this time a QI manager [hereon; Q1] came in. Q1 looked at the map and made a circumspect noise, which suggested to me that he felt the exercise wasn't taking shape in the way he hoped. So he took over and began with a new starting point, rather than starting from the beginning and saying ‘what happens when a patient enters the unit’, he flipped the process around and started with the desired outcome (the incentive scheme criteria for the discharge summary), and then asked the staff to think about the necessary steps that need to be in place in order to deliver this outcome. Q1 wrote down the incentive scheme criteria, and reminded everyone that 90% compliance was required, then wrote up a template set of prompts that needed to be answered at each stage of the map:

1. What needs to be done?
2. Who by?
3. What needs documenting?

Returning to the map, Q1 started with the note that said ‘bloods taken’:

Q: What happens after bloods are taken
A: The report comes back with the stage of AKI. Stage 1 or 2 are routine reports
Q: What happens to these?
A: The result goes on the EPR [electronic patient record]

Q1 puts a note up saying ‘AKI result on EPR’ and next to it puts a bright yellow note saying ‘incentive scheme (1)’.

Q: What happens to the 3s?
A: Flagged for biochemistry
Q: What does that mean?
A: The result is telephoned through to biochemistry
Q: Who answers the phone?
A: There isn't a dedicated member of staff to answer the phone
Q: What if no one answers?
A: We try again

Q1 puts a bright red note next to ‘flag for biochemistry’ which says: ‘Risk: delay, telephone unmanned’, then returns to the previous stage:

Q: AKI result on EPR – who is aware of this? Are people looking for AKI results? (Another ‘risk’ note goes up)

This kind of dialogue continued for approximately 20 minutes, by which time there were several ‘risk’ notes. Many of these related to the transfer of information between staff, and the actions which were prompted on the basis of information. Information transfer seemed to happen as part of the ‘everyday’ and it was not easy to systematise in the way the process mapping demanded. Where an action was required, there were usually several possible actions that could be taken depending on other variables. In these instances Q1 prompted for further information, and created new notes with more ‘risks’ as well as ‘actions’ required in order to improve the automatic prompts and flags that could appear on the EPR, in order to guide decision‐making. Q1 finished by asking the divisional manager to lead a process whereby all risks were collected together and an action plan made for each one. Q1 reminded everyone to think about the standard that is required (the incentive scheme and 90% compliance) and the three prompts that need to go with each action.

Process mapping mobilises a metaphor of the map and the journey which attempts to render events in a topographical and linear format. In the above encounter, the messy and tacit everyday process challenged this form of representation. Upon taking over, Q1 inverted the process, instead of starting with the patient and their entry to the unit, it now started with the desired outcome. To continue the metaphor of a journey, the mapping began with the destination and limited all possible routes according to this. This move naturalised the metaphor of the linear journey and instead made the everyday tacit process problematic. The actualisation of problems was documented according to specific ‘risks’ and ‘actions’ required for their amelioration. The process then created the possibility of ordering and ranking different clinical areas according to ‘riskiness’. It also highlighted areas in which more specific prompts for action were required. This brings us to our focus in the next section on the devices which could translate the process of ‘representing and intervening’ that we have just described into subsequent action. The inversion involved in this interrogation of practice according to a naturalised future becomes an important adjunct to financialisation, to which we will return in the final empirical section.

### Making caring practices count

In the process mapping, a situation was made problematic when the transfer of information and actions following that transfer were too dependent on the situated judgement and interaction of individuals. One of the tasks of the QI programme was to reduce the dependence on human initiated data generation and transfer. There were two technologies that were central to this attempt; the algorithm producing an electronic alert for AKI, and the EPR.

When routine blood work was sent to pathology, the algorithm produced an AKI alert if there was a measurable change in the levels of serum creatinine, a by‐product of normal muscle metabolism, from baseline – the degree of change is represented as AKI stage 1, 2 or 3. The baseline is taken from previous blood results. Only if there is a previous blood result on record can the calculation be made and the alert produced. Where there is no prior result recorded staff are required to find a baseline by searching manually through a patient's notes and following up with either a hospital where they have previously been admitted, or with their General Practitioner (GP), or by estimating on the basis of population data.

In addition to this reliance on data which might not be there, the algorithm also relies on human interaction and interpretation. It alerts staff only to the presence of an individual who is ‘at risk of’ developing AKI stage 1, 2 or 3. The alert does not substitute human work but requires and directs it. Qualitative judgement is required to interpret this result in the context of the specific circumstances of the case, and to make an appropriate decision. Because of the reliance on the baseline data, and the syndromic nature of AKI, the alert is a crude judgement, and is associated with both the problem of over and under alerting.

The imprecision of the technology, the dependence on pre‐existing data and the qualitative judgement of professionals demonstrate the enmeshing of technology with people that Mol (2008) describes as central to a logic of care, which must allow both the subjective experience of the patient and the situated judgement of the professional to ‘tinker’ in the decision‐making process. However, the ideal of automation inscribed in the algorithm can render problematic this ‘qualculative’ enmeshing of qualitative and quantitative information (Moser and Law 2006). Once the number is produced it enacts a binary – an individual either has AKI or they do not. This binary then produces two possible and divergent courses of action for staff. Each alert is documented and counts towards the statistical record of instances of AKI both within the hospital and nationally, while each instance of ‘not AKI’ is left undetected and not subject to an alert, with additional human resources required to investigate whether it might still be a case or not. The binary assumptions of calculative logics proliferate, with one binary producing another set: ‘AKI/Not AKI’ is followed up by ‘appropriate/inappropriate’ actions to be taken by staff (c.f. Peerally *et al*. 2017).

When the algorithm detects a possible AKI, the alert also registers instantaneously as a ‘banner’ on the EPR. This means that every worker looking at a particular individual's record will see a highlighted bar at the top of the screen alerting them to the possible presence of AKI. Along with the banner comes a set of prompts.

So I think the EPR keeps it in focus. So I think it's a kind of something that underpins the education support, the awareness raising. So I think that because it's there and coming at you every time you're looking at a patient that's kind of reinforcing us. So it's kind of triggering off those memories about, “oh yeah, well we did the AKI learning package the other week and now I know what that means and what that's all about.” So I think the fact that it is high profile within EPR and, you know, there is no kind of avoiding that (Q3).

It follows that blood that does not prompt an alert will make no further appearance on EPR, until such time that another sample is taken and the status changes. Blood that is marked as AKI becomes a target for communication and intervention, and the EPR ‘triggers’ the appropriate staff response. This reinforces the binary function of the algorithm, which reduces the complexity of qualitative decision‐making down to a 1/0 choice. The EPR then directs the appropriate steps to be taken for the ‘1s’. Together, the algorithm and the EPR then signal the point at which the number becomes detached from one normative order and becomes an active part of another; prompting a surveillance regime and directing particular actions, regulating responses and tallying the normal and deviant:

So if you're making a decision, so like the pharmacist's review, so the pharmacist's review conducted within 24 hours of admission or 24 hours of the first AKI alert, when you do that review there's a section on their review document that says; “I've discussed this with the clinical team”, “I've discussed this with … ” and you put the names of the people that you've discussed this with, and; “the actions we've agreed on are … ” and so on and so forth. So that will give you then a kind of audit trail of, “what did we do?”, “who did we communicate that with?”, “what actions did everybody take?”, so we can actually look at that pathway and say, “did we tread the right path for this patient?”, “did we do everything we possibly could, everything that best practice dictates we should and are we compliant with the guideline at the same time?”(Q3)

In the above quote the EPR plays a role in developing the relationship among care, risk and audit. Organised care is represented as risky (as in the example of process mapping above). The EPR also conducts a kind of implicit problematisation, but in addition offers a more directive means to ‘improve’ care, by producing an auditable account of the ‘right path’. The understanding of ‘good care’ inscribed in the EPR is therefore ‘compliant’ care. The implication is that if something goes wrong, an account can be presented through which fault, or its absence, can be identified. A further implication is that this kind of ‘process’ data can be collected together to tabulate and compare the performance of departments within an organisation, or between different organisations. In the last section of our findings, we show how the incentive scheme acts as a mechanism to translate the risks associated with these data into costs.

### Decision‐making using kidney numbers

Once practices and the organisational, human and relational processes in which they are situated have been materialised into numbers, these numbers participate in new ways in the experience out of which they were generated. The adoption of the incentive scheme within the hospital provides the empirical material to demonstrate this process. The incentive scheme was adopted in April 2015, after which there was a 3‐month period during which the organisation agreed thresholds with NHS England against which their data would be monitored. Data were then counted over four quarters, with the threshold rising up to the pre‐agreed limit by the fourth quarter. The agreement of thresholds is therefore a crucial part of the process, and these considerations were high on the agenda of the AKI steering group over this period:

#### Steering Group May 2015

Q2 is taking the group through the ‘assurance’ part of the agenda, which at the moment is dominated by the incentive scheme. Q1 is concerned that the AKI stages are not being recorded, he had thought that EPR should improve this, but so far it doesn't seem to have (he notes this with the EPR technician, to be discussed in more detail later on), he also notes that medication reviews are poor (I think he means not being done when they should be, rather than being done badly), and that there are missing discharge summaries (It is not clear whether this means the whole discharge summary is missing, or just the AKI data that is meant to be recorded on them). Q2 ends with a warning that July will be the start of financial penalties if they don't meet the standard. She sets priorities: EPR and medication review.

#### Steering Group June 2015

The agenda today is almost entirely assurance (incentive scheme). Q2 once again leads the discussion, though this time the lead renal consultant (RC1) also contributes. RC1 sets out the pre‐agreed thresholds across the four quarters. RC1 emphasises the importance of ‘aiming low’ in the thresholds, there are some smiles around the room at this, but it appears that RC1 is being quite sincere, saying that if the organisation don't comply with these thresholds then they will face financial penalties. RC1 finishes by saying that data ‘counts’ from the 1^st^ July (next week).

In the above excerpts, the incentive scheme directs practice through ‘thresholds’ and ‘penalties’: thresholds are a conservative guess at what might be achieved in practice. As observed above, practice is made up of a number of interacting qualitative and quantitative processes, all with multiple contextually sensitive variables. Once the threshold is set, the quantitative target directs the practice. Improvements in practice are then driven by the idea of ‘penalties’, which associates insufficient improvement with cost. This involves the naturalisation of the outcome yet to be achieved – that is, it creates a projection in which the full ‘bonus’ that the incentive scheme represents is achieved, and makes this the benchmark against which the present is costed.

In the example of process mapping above, we saw a compressed example of this future inversion in practice, when Q1 used the incentive scheme standards as a means to render a simplified representation of the hypothetical patient journey, in so doing highlighting areas of risk. In the working group meetings that followed the initiation of the incentive scheme, this process was stretched out over a period of months, with each new set of numbers prompting the further identification and interrogation of problems in practice:

#### Steering Group Memo December 2015

The AKI incentive scheme figure is currently at 78% and our target is 90% by the end of Quarter 4 (March 16). There is concern that some patients are still falling through the net at weekends when discharge information is not updated on EPR. It may be worth looking at the work done by the surgical high dependency unit around out of hour's interventions (urine dip/protein).

There needs to be exploration about whether EPR could accommodate a drop down option for the different repeat blood options we devise with the junior doctors.

Educating junior doctors is key to getting this right. We will be talking to Foundation Year 1s every 2 weeks prior to their Tuesday teaching session.

#### Steering Group Memo March 2016

We were at 75% in Q3 against a target of 70% and need to reach 90% at Q4.

The focus is on discharge summaries and the QI team have informed all medical directors.

There are a few things we could look at:

1. Pharmacy – EPR might miss an opportunity when there is a flag after the follow‐up is completed.
2. Consultant X's algorithm on bloods at discharge has been sent to junior doctors and pharmacology and is being tested.
3. Query to Q3 re showing the AKI has been resolved on EPR.
4. “Bloods” within 1 week is unlikely to be done by GPs and the incentive scheme asks for a “Reasonable Follow‐up”.

As these two excerpts show, the data, whether the organisation is meeting the quarterly targets or not, are taken as an opportunity to further interrogate practice. Qualitative judgement is then mobilised to suggest further possibilities for the regulation of moment‐to‐moment action. Often this revolves around ‘known’ risks (such as junior doctors, who rotate regularly between departments, or situations involving the transfer of information or individuals between departments, such as the pharmacy reviews). This search for possible further regulation then drives the selection and presentation of data to be shown in EPR, and further develops the directives that are deemed necessary to prompt action towards compliance in the moment.

The incentive scheme prompts the organisation to make a set of financial projections, which are objectified and turned back onto practice in order to direct improvement. This process renders risks as costs – the compliance inscribed in the EPR here is transformed into financial compliance.

The incentive scheme projected a financial future which established a relationship of debt within the programme – if the programme was not successful then the organisation would be penalised. Therefore, not only can the incentive scheme be shown to be directing the QI programme, but in so doing, it created a separation of orders, where the ‘narrative’ of the QI programme became a speculative attempt to explain the numbers and a performative account to maintain commitment on the frontline, while the numbers were detached in order to be made public through regional and national reporting networks.

## Discussion

The financialisation of AKI involves the transformation of discrete episodes of illness, and their associated practices, into numbers on an accounting table. This results in a schism, in which the numbers of improvement become the means by which organisations can present an external account to be tabulated and compared with other organisations, while the QI programme itself becomes a performative narrative to maintain commitment and stability on the frontline (c.f. Froud *et al*. 2006). Our argument is significant for the study of healthcare organisation and points the way to a future research agenda for the study of financialisation in this and other public sector domains.

In the first presentation of data, we used the example of process mapping to show how the wards participating in the QI programme were represented as problematic and made a target for intervention. In this case, the first step towards the generation of a new normative order involved a *disordering* of the present. In the second presentation of data, we showed how the algorithm and EPR worked together to enact a shift from one (qualitative) order to another (calculative). This created an auditable account of a compliant care process and directed staff actions towards the achievement of it. In the last presentation of data, we showed how the incentive scheme translates compliance into finance, by projecting the future attainment of a financial goal back upon practice. In so doing it establishes a debt relation between the programme and the organisation. In this way financially oriented forms of accountability such as the incentive scheme ‘hinge’ between government debt and the daily practices of care within hospitals (c.f. du Gay *et al*. 2012). The economic and political narrative of austerity then directs the production of numbers at the organisational level for the purposes of finance and accounting.

Our study contributes to the emerging body of work on financialisation in the public sector (Froud *et al*. 2009, du Gay *et al*. 2012). Our aim has been to describe the materialisation of financial concerns and relationships within the banal everyday of a QI programme for AKI. As quality, safety and improvement are key everyday operators within all public service organisations, we expect our argument to be broadly generalisable to other domains within and beyond health care and in other national contexts. Of course, the mechanisms and the translational work they do will be key to specifying these processes within different contexts.

Within the domain of sociological studies of healthcare quality and safety, we have built upon Waring's (2009) study of the translation of qualitative judgement into quantitative organisational arrangements (from care to risk), by showing how these arrangements then go to work, both back ‘down’ upon daily care and ‘up’ to organisational accounting and decision‐making (from risk to cost). This then demonstrates how financialisation moves us beyond the familiar tensions of managers versus professionals, as the work of both ends up being subjugated by an organisational need to produce improved numbers. This could be read as the organisational dimension of the process Waring (2007) describes as adaptive regulation, in which medical professionals internalise managerial safety norms even as they resist them. Here, beyond both managers and professionals, the idea of ‘safety’ itself was being regulated by financial norms, and although it was often clear that individuals were aware of this regulation and its drivers – for example, in the widespread criticism we heard of the incentive scheme, from both managers and practitioners, and its directive and financialised approach – this did not stop individuals directing their energies towards the successful achievement of the standards it required (cf. Allen 2016), or prevent the co‐optation of a set of medical practices by another order (cf. Harrison 2009). This indicates an implicit collective understanding that the macroprocesses described in the early part of this study are treated as inevitable, and a kind of work we can refer to as *organisational caretaking* is directed to ensure organisational survival. However, this very same work further reproduces and naturalises those same conditions. Therefore, as the numbers produced by this work appear increasingly independent, so too the importance of adaptation, resistance and other kinds of human agency are diminished.

## Conclusion

Our analysis has extended the literature on financialisation to open up a new way of looking at healthcare organisation. Almost half a century after the introduction of new public management was justified with reference to the need for entrepreneurialism, individuals within public service organisations continue to live with, enact and internalise the logics of markets and managerialism. Producing auditable accounts of care, reducing costs and limiting human autonomy now appear as matters of fact.

Among the possible consequences of the transformation we have described here is the neglect of what Mol (2008) describes as the *enmeshed* and *entangled* practice of care. This kind of practice is essential for the increasingly complex meeting of subjectivity, technology and politics experienced in contemporary healthcare organisation. Financialisation can therefore threaten health care. Caring practices in health will not simply stop. Human agency and the professional ethos continue to be important, but they are increasingly being rendered organisationally illegitimate, wasteful, risky and costly through financialisation. This is compounded by further decreases in public expenditure on health care, which threaten the ability of staff to ‘go beyond’ in the manner that care and their professional norms demand. Possible solutions to this problem are beyond the scope of this study but problematising the present, as we have, is a helpful starting point for thinking about how to generate alternative futures *in* the present.
